# The Rising Burden of Salmonellosis Caused by Monophasic *Salmonella* Typhimurium (1,4,[5],12:i:-) in Greece and New Food Vehicles

**DOI:** 10.3390/antibiotics10020185

**Published:** 2021-02-13

**Authors:** Georgia Mandilara, Theologia Sideroglou, Anthi Chrysostomou, Iliodoros Rentifis, Theofilos Papadopoulos, Michalis Polemis, Myrsini Tzani, Kyriaki Tryfinopoulou, Kassiani Mellou

**Affiliations:** 1 National Reference Centre for Salmonella, Faculty of Public Health Policies, School of Public Health, University of West Attica and Athens, 12243 Egaleo, Greece; gmandilara@uniwa.gr (G.M.); iliodrent82@hotmail.com (I.R.); 2 Department of Foodborne and Waterborne Diseases, National Public Health Organization, 15123 Athens, Greece; t.sideroglou@eody.gov.gr (T.S.); a.chrysostomou@eody.gov.gr (A.C.); 3 European Programme for Intervention Epidemiology Training (EPIET), European Centre for Disease Prevention and Control, (ECDC), 17165 Stockholm, Sweden; theofilos23@vet.auth.gr (T.P.); mtzani@minagric.gr (M.T.); 4 Department of Epidemiology and Public Health, Sciensano, 1050 Brussels, Belgium; 5 Central Laboratory of Public Health, National Public Health Organization, Vari, 16672 Attica, Greece; mixalispolemis@gmail.com (M.P.); k.tryfinopoulou@eody.gov.gr (K.T.)

**Keywords:** *Salmonella*, monophasic Typhimurium, outbreak, multiresistant

## Abstract

Monophasic *Salmonella typhimurium* is of increasing importance worldwide. Here we present the available data regarding monophasic *S. typhimurium* from 2007 to 2019 in Greece, in order to assess its public health impact. Surveillance data, data on antimicrobial resistance, molecular typing by pulsed-field gel electrophoresis (PFGE), and results of the investigation of monophasic *S*. *typhimurium* outbreaks were analyzed. Overall, 403 cases were identified; 329 (81.6%) sporadic and 74 (18.4%) related to two community outbreaks in 2017. A total of 305 isolates from sporadic cases tested for antimicrobial resistance revealed resistance to ampicillin, streptomycin, sulphamethoxazole, and tetracycline (41.3%). Some 23.3% were further resistant to trimethoprim and 5.2% were also resistant to chloramphenicol. Outbreak 1 in 2017 with 37 identified cases was attributed to the consumption of raw milk from a vending machine and isolates were resistant to ampicillin, streptomycin, sulphamethoxazole, tetracycline, and trimethoprim. Outbreak 2 also with 37 cases was attributed to the consumption of pork and isolates were resistant to the five above mentioned antibiotics plus chloramphenicol. The number of human monophasic *S*. *typhimurium* isolates is low; however, since 2009, it has been among the five most frequently identified serotypes in Greece. Investigation of the outbreaks revealed that other vehicles apart from pork may be implicated in the occurrence of outbreaks.

## 1. Introduction

Salmonellosis is a foodborne disease caused by the bacteria *Salmonella* that results in a considerable global burden of morbidity and mortality [1]. The public health impact of *Salmonella* is aggravated by antimicrobial resistance (AMR) [2]. There are over 2500 serotypes of *Salmonella* that have been reported worldwide; *Salmonella enterica enterica* serotypes enteritidis and typhimurium have been reported to be the most common causes of human salmonellosis [3,4].

Over the past half century, *Salmonella typhimurium* epidemiology has been characterized by successive waves of prevalent multi-resistance to antibiotics strains [5]. The prevalence of the multi-resistant monophasic variant of *Salmonella typhimurium* among human cases has increased considerably in many countries in the world over the last 20 years [6]. The monophasic variant *S*. 4,[5],12:i:- is antigenically similar to *S*. *typhimurium* but does not express the second-phase flagellar antigen, which is identified as 1,2 in the *S*. *typhimurium* antigenic formula [7].

Since the first variant of *Salmonella enterica* subspecies enterica serovar typhimurium with antigenic structure 1,4,[5],12:i:- (monophasic *S. typhimurium*), was identified in the late 1980s from poultry in Portugal [8], it has become one of the most common *Salmonella* serotypes in Europe and is considered to be among the three most common serotypes isolated from humans in EU since 2011 [4]. Different strains of monophasic *S*. *typhimurium* have emerged in various countries at different times, and applying phenotypic and molecular typing methods, these strains have generally been distributed in three prevalent clonal lines, the Spanish clone, the European clone, and the U.S. clone [9]. The multidrug resistant Spanish and European clonal lineages are also characterized by a high occurrence of genes encoding tolerance to copper, silver, and mercury [10]. The use of non-antibiotic compounds (heavy metals) with antimicrobial activity in animal husbandry in order to control foodborne pathogens and the use as growth promoters in pork production may lead to the prevalence of these lineages [11,12]. It seems that new monophasic *S. typhimurium* strains are continuously emerging from different *S*. *typhimurium* strains through different genetic events [9]. Still, the proportion of human cases due to this serovar is low (6.5% of all reported cases in 2019), with a mean notification rate of salmonellosis in the EU and EEA/EFTA countries to be 19.9 cases per 100,000 population for the year 2019 [13]. Based on the last published data from the European Food Safety Authority, monophasic *S*. *typhimurium* was associated mainly with pig (49.7%) and broiler sources (35.3%) [4]. Reporting of monophasic *S*. *typhimurium* was inconsistent in the past due to variability in the nomenclature used to report this serotype in many countries, resulting in many isolates being reported only as “Group B” or “Subspecies *enterica*” and some isolates being incorrectly reported as serotype Typhimurium [14].

The aim of the present study is to review the available epidemiological data on monophasic *S*. *typhimurium* since 2007 in order to assess the public health impact of this serotype in the country. We also present the results of the phenotypic and molecular characterization of monophasic *S. typhimurium* human strains, isolated in Greece, with respect to their antimicrobial susceptibility profile, and DNA fingerprinting using the pulsed-field gel electrophoresis (PFGE) method.

## 2. Results

### 2.1. General Results

**Figure 1:**
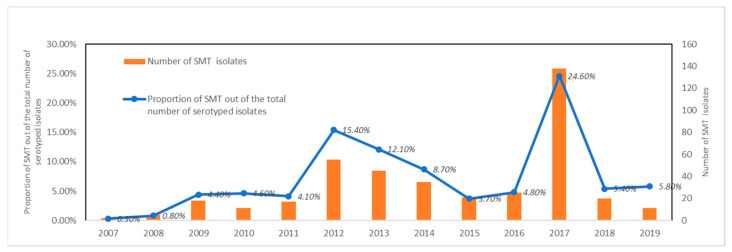
Human monophasic *Salmonella typhimurium* cases and proportion of the total number of Salmonella isolates, Greece, 2007–2019.

Overall, 402 cases were identified; 329 (81.6%) were considered sporadic and 74 (18.4%) were related to two community outbreaks in 2017. The median age of cases was 6 years (range 0–85 years) and information regarding gender was available for 392 cases (210 were male—53.6%). The median number of reported cases of monophasic *S*. *typhimurium* for the years 2007–2019 was 20 (range 2–138). Isolation rate ranged from 0.3% in 2007 to 24.6% in 2017 with monophasic *S. typhimurium* ranking at the moment among the five most frequently identified serotypes in the country. From 2007 to 2019, the mean frequency of isolation was 7.3 (SD = 6.4) (Figure 1).

Time series analysis did not show any statistically significant time trend of the number of monophasic *S*. *typhimurium* isolates (*p-value* = 0.066).

The mean age of outbreak 1 and outbreak 2 related cases was 6.2 years (SD: 4.02) and 24.8 years (SD: 23.3), respectively. The mean age of sporadic monophasic *S*. *typhimurium* cases was 15.6 years (SD: 1.51), significantly lower than the mean age of the overall sporadic salmonellosis cases identified in the same period (*p-value* = 0.0015). Reported symptoms of sporadic and outbreak related SMT cases are similar to the ones reported for the rest of Salmonella serotypes, however bacteremia was reported less frequently among monophasic *S*. *typhimurium* cases (*p-value* = 0.002).

### 2.2. Susceptibility Testing of Sporadic Cases, 2007–2019

**Figure 2:**
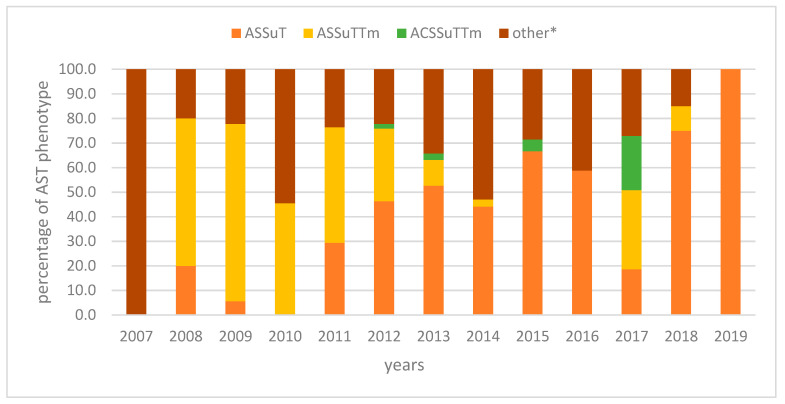
Percentage of human monophasic *S*. *typhimurium* isolates with the ampicillin–streptomycin–sulfonamide–tetracycline (ASSuT), ampicillin–streptomycin–sulfonamide–tetracycline-trimethoprim (ASSuTTm), and ampicillin–chloramphenicol-streptomycin–sulfonamide–tetracycline-trimethoprim (ACSSuTTm) resistance phenotypes, Greece, 2007–2019. other* AST phenotypes (T, SSuT, ASuT, ASSuTm, S, AS, A, STm, Tm, CSu, Su).

In 2007–2019, 305 monophasic *S*. *typhimurium* isolates from sporadic cases were tested for antimicrobial resistance. Of these, 126 (41.3%) were resistant to ampicillin, streptomycin, sulphamethoxazole, and tetracycline (ASSuT) (two of them produced Extended Spectrum b-lactamases-ESBL), 71 (23.3%) were further resistant to trimethoprim (ASSuTTm), and 16 (5.2%) also to chloramphenicol (ACSSuTTM); 92 (30.2%) isolates presented a variety of other AST phenotypes in low frequencies (T, SSuT, ASuT, ASSuTm, S, AS, A, STm, Tm, CSu, Su in) (Figure 2).

### 2.3. Molecular Typing by Pulsed Field Gel Electrophoresis, 2018–2019

**Figure 3:**
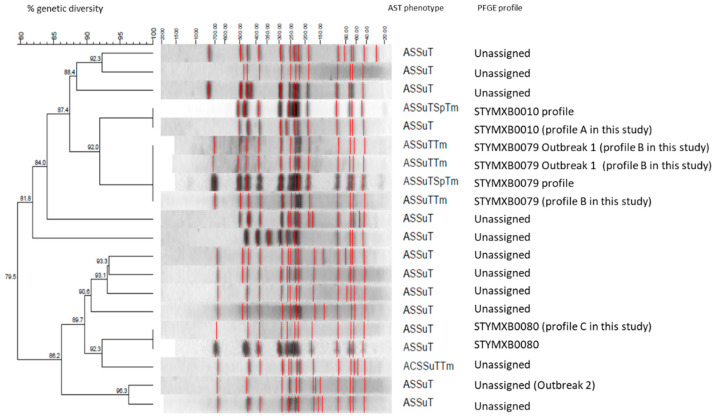
Dominant pulsed field gel electrophoresis profiles identified in human isolates determined as monophasic *S*. *typhimurium* in Greece, 2018–2019 and in human isolates of the two outbreaks in 2017. *Salmonella* Braenderup H9812 was used as reference strain. (Profiles A, B, and C were assigned with reference strains of ECDC-EFSA molecular typing database).

XbaI PFGE analysis of the 29 monophasic *S*. *typhimurium* sporadic isolates (human isolates of period 2018–2019) and of the 74 isolates of the outbreak cases revealed over 15 different profiles (Figure 3). Among the sporadic isolates, profile STYMXB0080 was the predominant (8/29, 27.5%), followed by STYMXB0010 (6/29, 20.7%) and STYMXB0079 (2/29, 6.9%).

### 2.4. Outbreaks

In both monophasic *S*. *typhimurium* outbreaks, cases had initially been classified as sporadic since they had reported no contacts with other gastroenteritis cases and were geographically scattered without an apparent epidemiological link to other cases or a specific food vehicle. All cases regarded Greek citizens, without travel history abroad.

#### 2.4.1. Outbreak 1

In outbreak 1, a cluster of 37 monophasic *S*. *typhimurium* isolates resistant to ampicillin, streptomycin, sulphamethoxazole, tetracycline, and trimethoprim (ASSuTTm) was identified in June 2017. Isolates belonged to the PFGE profile Xba0079 (Figure 3). The median age of cases was 5 years (range: 1–17) and 26 (68.4%) of the cases were males. Cases reported date of symptoms onset between March and July.

Based on the results of the 1:1 unmatched case-case study performed (using salmonellosis cases with a positive culture for a serotype other than monophasic *S*. *typhimurium* as a comparative group), consumption of raw milk from a vending machine of a specific company was the only exposure that had a statistically significant association with disease occurrence (OR: 51.0, 95% CI: 3.79–2359.02). Overall, 17 of the 20 outbreak cases reported consumption of milk of the same origin.

The Hellenic Food Safety Authority inspected the premises and checked the HACCP (Hazard Analysis and Critical Control Points) system of the implicated—from the epidemiological investigation—dairy company for critical points that required the implementation of correction measures. Five milk samples from different points of purchase were collected in July and tested negative for *Salmonella* spp. at the Central Public Health Laboratory. After this outbreak, official controls were intensified in such companies and controls of the milk automated vending machines were included in the official control program of the National Food Safety Authority in Greece.

#### 2.4.2. Outbreak 2

In August 2017, a second cluster of monophasic *S*. *typhimurium* isolates resistant to ampicillin, chloramphenicol, streptomycin, sulphamethoxazole, tetracycline, and trimethoprim (ACSSuTTm) was identified. Isolates presented an indistinguishable PFGE profile (unassigned) (Figure 3).

Overall, 37 cases were identified. The median age of cases was 11.5 years (range: 1–79) and (50%) of the cases were males.

Again, a 1:1 unmatched case-case study was performed using salmonellosis cases with a positive culture for a serotype other than monophasic *S*. *typhimurium* as a comparative group. In the logistic regression analysis, outbreak cases were 22 (95% CI: 1.2–394) times more likely to have consumed pork in the three days before symptoms onset than sporadic cases.

During the environmental investigation of the outbreak, public health authorities inspected the restaurants that were mentioned by two or more outbreak cases and took environmental samples from pig farms and from food processing facilities linked with these restaurants. They examined preparation processes for the foods served, and reviewed the order and delivery books of the restaurants. The ingredients of incriminated foods were identified and traced to their sources (food processing facilities and farms). Food specimens from the days of the symptoms’ onset were no longer available when the investigations commenced. Tracing identified one common supplier of pork, a slaughterhouse and a pig farm. Samples taken from a common supplier found among two restaurants, a pig farm, a commercial pig herd (fecal, dust samples were taken), and the ileocecal lymph nodes taken from swine at the corresponding slaughterhouse were tested to assess the status of Salmonella infection in pigs bred and slaughtered in the prefecture. Collected samples tested negative for Salmonella spp.

## 3. Discussion

In Greece, after monophasic *S. typhimurium* was first identified in 2007 [15], the isolation rate increased but remained low in the following years. Since then, it has been among the five most common identified *Salmonella* serotypes [16,17]. The emergence of this serotype seems to cover the niche from the decrease of *S*. *enteritidis* and *typhimurium*, a finding also documented in other countries [18,19]. In European Union member states, monophasic S. 1,4,[5],12:i:- accounted for almost 7% of total human salmonellosis cases reported yearly for the last decade and ranked third among the most reported Salmonella serotypes [4]. No outbreaks were documented until 2016 and the epidemiological situation was similar to the other European countries.

The lower age of the cases caused by monophasic *S. typhimurium* compared to the rest of salmonellosis cases may reflect milder disease as also indicated by their lower proportion of invasive disease. As a result, adults may not seek medical attention as much as in other salmonelloses. Differences in the severity of disease among different *Salmonella* serotypes have been reported in the literature [20].

Our data revealed that different strains of monophasic *S*. *typhimurium* circulate in Greece. The multi-resistant phenotype of ASSuT accounted for 41.3% of monophasic *S*. *typhimurium* between 2007–2019. ASSuTTm was more prevalent until 2011 in human isolates in Greece [15], and it was not observed at all in 2019 isolates. Moreover, several other strains are circulating with different AST phenotypes. The major European clone from 2005 (ASSuT phenotype, RR1–RR2/RR3 resistance regions, sequence type ST34) has spread in several countries across the European Union [7,18,21,22] and as other studies support, European clone seems to be slowly overtaking the Spanish and U.S. clones as the most prevalent [23]. Further comparative whole genome sequencing and phylogenomic analysis are needed in order to determine whether the Greek monophasic *S.* Typhimurium isolates with ASSuT resistance phenotype belong to the European clone.

ESBLs were detected in two monophasic *S*. *typhimurium* isolates. The presence of ESBLs in monophasic *S*. *typhimurium* human isolates have been reported previously in several countries, though in only few isolates [24,25,26]), indicating however the need for enhanced surveillance of this serovar in terms of acquiring and spreading ESBLs genes. Molecular typing by PFGE revealed a variety of different profiles, with STYMXB0080 being the predominant. According to previous study for the period 2007–2011 STYMXB0010 was the most frequent profile in human isolates and STYMXB0080 was only observed in food isolates [15]. The antimicrobial resistance phenotype and PFGE profiles in the country are compatible to the ones previously described in Greece and in other European countries [18]. The results of the study support that monophasic *S*. *typhimurium* is capable of spreading unique strains and diverse clones with broad antibiotic resistance in different areas [18,27].

The identification of the two outbreaks of multi-resistant monophasic *S*. *typhimurium* in 2017 depicted the importance of laboratory surveillance of salmonellosis for the identification of community outbreaks that cannot easily be identified via the mandatory notification system. The availability of the antimicrobial resistance profiles is essential, not only for clinical reasons, but also for improving identification of outbreaks. Additionally, subtyping using PFGE is a very good method to distinguish clones of this particular serovar as it seems to be very diverse [28].

The two distinct community outbreaks in 2017 were identified at the same time that the number of reported cases at a European level remained at the same levels as in 2015 and 2016 [2].

In one of the outbreaks, the epidemiological investigation showed that pork was the vehicle of transmission, a finding compatible with the fact that pigs are considered the main animal reservoir for monophasic *S*. *typhimurium*. This is a very well documented risk factor [29,30]. However, our data suggested that other food vehicles can be implicated with the occurrence of outbreaks, such as milk from self-service vending machines. Several outbreaks related to such machines in the previous years have been reported [31].

As a conclusion, the available data in Greece support that monophasic *S*. *typhimurium* are emerging with an increasing antimicrobial resistance phenotype. The burden of the disease may potentially increase in the following years and clinical implications for treatment of salmonellosis should be taken into account. Public health authorities should be prepared to take measures and investigation should also focus to vehicles other than pork. Future studies could evaluate whether isolates from both outbreaks belong or not to a single clade within the Greek monophasic *Salmonella typhimurium* population and also test for coexistence of resistance to heavy metals, as described in other countries.

## 4. Methods

### 4.1. Data Sources

Human salmonellosis is a mandatory notifiable disease in Greece. All diagnosed cases are reported to the National Public Health Organization (NPHO) which manages the surveillance of infectious diseases in the country.

*Salmonella* isolates are sent to the National Reference Laboratory for *Salmonella* for serotyping and antimicrobial susceptibility testing. Each isolate is accompanied by a short form that includes the name and demographic characteristics (age, sex, date of birth, region) of the patient, type of specimen, and date of specimen collection. Results of serotyping and antimicrobial susceptibility testing are shared with the laboratories and NPHO. Data are recorded in the Epidata Manager statistical package.

Cases are classified as sporadic and outbreak-related. Definition of sporadic and outbreak-related cases is based on the collected epidemiological data. A case is recorded as sporadic when it has no apparent epidemiological link with other cases and as outbreak-related when the available data suggest an epidemiological link.

Data collected during outbreak investigations (epidemiological, laboratory, and environmental) are recorded in a separate specially designed database. Demographic data, age, sex, geographical distribution of cases, results of analytical studies conducted, laboratory results, and implemented control measures are recorded.

Data from both databases were analyzed using Stata v12. Quantitative variables are presented as means ± standard deviations or as medians and interquartile ranges (IQR) and qualitative variables as absolute frequencies and percentages. Interrupted time series analysis was performed on the annual number of isolates from 2007 to 2019 to assess temporal trend (negative binominal regression model).

### 4.2. Laboratory Methods

#### 4.2.1. Serotyping

Serotyping for the identification of somatic antigen O and flagellar antigens H (phase 1 and 2) is performed by the slide agglutination method according to the White–Kaufmann–Le Minor Scheme [32]. To confirm that strains serotyped as *S*. serovar 1,4,[5]:i:- are indeed monophasic *S. typhimurium*, one multiplex PCR assay is applied to detect the presence of the specific for *S. typhimurium* IS200 fragment, and the phase 2 (fljB) flagellar antigen gene [33].

#### 4.2.2. Susceptibility Testing

Susceptibility testing is performed by the agar disk diffusion method (Kirby–Bauer) according to the protocols and guidelines of the European Committee on Antimicrobial Susceptibility Testing—EUCAST [34]. The following antibiotics (Oxoid) are tested; ampicillin (A), amoxicillin-clavulanic acid, ceftazidime, cefotaxime, ciprofloxacin, chloramphenicol (C), tobramycin, netilmicin, nalidixic acid (Na), pefloxacin, streptomycin (S), sulphamethoxazole (Su), tetracycline (T), trimethoprim (Tm), and sulfamethoxazole-trimethoprim. Phenotypic confirmation of Extended Spectrum b-lactamases was performed according to EUCAST guidelines using the double-disk synergy test (DDST) [35].

#### 4.2.3. Molecular Typing by Pulsed Field Gel Electrophoresis

PFGE is performed after digestion of genomic DNA with XbaI according to the Pulse-Net protocol [36]. Fingerprints are analyzed using Bionumerics 6.6 (Applied Maths). Dendrograms are constructed using the Dice similarity coefficient and the unweighted pair group method with arithmetic averages (UPGMA), with optimization and position tolerance set at 0.5% and 1.5%, respectively. PFGE was performed for sporadic human isolates from 2018–2019 and for outbreak strains in 2017. In Greece, molecular typing using PFGE method is only applied when investigating a possible outbreak, in order to enhance or not the outbreak scenario. However, since two monophasic *Salmonella typhimurium* outbreaks were observed in 2017 it was decided to monitor this certain serotype using PFGE, in 2018 and 2019.

## Data Availability

The data presented in this study are available on request from the corresponding author.

## References

[1] World Health Organization—WHO Estimates of the Global Burden of Foodborne Diseases Foodborne Diseases Burden Epidemiology Reference Group 2007–2015. https://apps.who.int/iris/handle/10665/199350.

[2] Baker S., Thomson N., Weill F.X., Holt K.E. (2018). Genomic insights into the emergence and spread of antimicrobial-resistant bacterial pathogens. Science.

[3] Center for Disease Control and Prevention National Enteric Disease Surveillance: Salmonella Annual Report. https://www.cdc.gov/nationalsurveillance/pdfs/2016-Salmonella-report-508.pdf.

[4] European Food Safety Authority & European Centre for Disease Control and Prevention (2019). The European Union One Health 2018 Zoonoses Report. EFSA J..

[5] Rabsch W.T.S., Windhorst D., Gerlach R.G. (2011). Typing Phages and Prophages of Salmonella.

[6] Soyer Y., Moreno Switt A., Davis M.A., Maurer J., McDonough P.L., Schoonmaker-Bopp D.J., Dumas N.B., Root T., Warnick L.D., Grohn Y.T. (2009). Salmonella enterica serotype 4,5,12:i:-, an emerging Salmonella serotype that represents multiple distinct clones. J. Clin. Microbiol..

[7] Hopkins K.L., Kirchner M., Guerra B., Granier S.A., Lucarelli C., Porrero M.C., Jakubczak A., Threlfall E.J., Mevius D.J. (2010). Multiresistant Salmonella enterica serovar 4,[5],12:i:- in Europe: A new pandemic strain?. Euro Surveill.

[8] Machado J., Bernardo F. (1990). Prevalence of Salmonella in chicken carcasses in Portugal. J. Appl. Bacteriol..

[9] Arrieta-Gisasola A., Atxaerandio-Landa A., Garrido V., Grillo M.J., Martinez-Ballesteros I., Laorden L., Garaizar J., Bikandi J. (2020). Genotyping Study of Salmonella 4,[5],12:i:- Monophasic Variant of Serovar Typhimurium and Characterization of the Second-Phase Flagellar Deletion by Whole Genome Sequencing. Microorganisms.

[10] Mourao J., Novais C., Machado J., Peixe L., Antunes P. (2015). Metal tolerance in emerging clinically relevant multidrug-resistant Salmonella enterica serotype 4,[5],12:i:- clones circulating in Europe. Int. J. Antimicrob. Agents.

[11] Clark C.G., Landgraff C., Robertson J., Pollari F., Parker S., Nadon C., Gannon V.P.J., Johnson R., Nash J. (2020). Distribution of heavy metal resistance elements in Canadian Salmonella 4,[5],12:i:- populations and association with the monophasic genotypes and phenotype. PLoS ONE.

[12] Mastrorilli E., Pietrucci D., Barco L., Ammendola S., Petrin S., Longo A., Mantovani C., Battistoni A., Ricci A., Desideri A. (2018). A Comparative Genomic Analysis Provides Novel Insights Into the Ecological Success of the Monophasic Salmonella Serovar 4,[5],12:i. Front. Microbiol..

[13] European Centre for Disease Control and Prevention Surveillance Atlas of Infectious Diseases. https://atlas.ecdc.europa.eu/public/index.aspx?Dataset=27&HealthTopic=46.

[14] European Food Safety Authority (EFSA) (2010). Scientific Opinion on monitoring and assessment of the public health risk of “Salmonella Typhimurium-like” strains. EFSA J..

[15] Mandilara G., Lambiri M., Polemis M., Passiotou M., Vatopoulos A. (2013). Phenotypic and molecular characterisation of multiresistant monophasic Salmonella Typhimurium (1,4,[5],12:i:-) in Greece, 2006 to 2011. Euro Surveill.

[16] Greek System for the Surveillance of Antimicrobial Resistance. http://www.mednet.gr/whonet/.

[17] Hellenic National Public Health Organization (Hellenic NPHO) Epidemiological Data on Salmonellosis in Greece, 2004–2019. https://eody.gov.gr/wp-content/uploads/2020/04/Epidemiological-data-for-Salmonellosis_2004-2019.pdf.

[18] Sun H., Wan Y., Du P., Bai L. (2020). The Epidemiology of Monophasic Salmonella Typhimurium. Foodborne Pathog. Dis..

[19] Bawn M., Alikhan N.F., Thilliez G., Kirkwood M., Wheeler N.E., Petrovska L., Dallman T.J., Adriaenssens E.M., Hall N., Kingsley R.A. (2020). Evolution of Salmonella enterica serotype Typhimurium driven by anthropogenic selection and niche adaptation. PLoS Genet..

[20] Jones T.F., Ingram L.A., Cieslak P.R., Vugia D.J., Tobin-D’Angelo M., Hurd S., Medus C., Cronquist A., Angulo F.J. (2008). Salmonellosis outcomes differ substantially by serotype. J. Infect. Dis..

[21] Mossong J., Marques P., Ragimbeau C., Huberty-Krau P., Losch S., Meyer G., Moris G., Strottner C., Rabsch W., Schneider F. (2007). Outbreaks of monophasic Salmonella enterica serovar 4,[5],12:i:- in Luxembourg, 2006. Euro Surveill.

[22] Dionisi A.M., Graziani C., Lucarelli C., Filetici E., Villa L., Owczarek S., Caprioli A., Luzzi I. (2009). Molecular characterization of multidrug-resistant strains of Salmonella enterica serotype Typhimurium and Monophasic variant (S. 4,[5],12:i:-) isolated from human infections in Italy. Foodborne Pathog. Dis..

[23] Elnekave E., Hong S., Mather A.E., Boxrud D., Taylor A.J., Lappi V., Johnson T.J., Vannucci F., Davies P., Hedberg C. (2018). Salmonella enterica Serotype 4,[5],12:i:- in Swine in the United States Midwest: An Emerging Multidrug-Resistant Clade. Clin. Infect. Dis..

[24] Rodriguez I., Jahn S., Schroeter A., Malorny B., Helmuth R., Guerra B. (2012). Extended-spectrum beta-lactamases in German isolates belonging to the emerging monophasic Salmonella enterica subsp. enterica serovar Typhimurium 4,[5],12:i:- European clone. J. Antimicrob. Chemother..

[25] Fernandez J., Garcia V., Bances M., Rodicio M.R. (2016). CTX-M-14 production by a clinical isolate of the European clone of Salmonella enterica 4,[5],12:i. J. Glob. Antimicrob. Resist.

[26] Wang Z., Xu H., Tang Y., Li Q., Jiao X. (2020). A Multidrug-resistant Monophasic Salmonella Typhimurium Co-harboring mcr-1, fosA3, bla CTX-M-14 in a Transferable IncHI2 Plasmid from a Healthy Catering Worker in China. Infect. Drug Resist.

[27] Petrovska L., Mather A.E., AbuOun M., Branchu P., Harris S.R., Connor T., Hopkins K.L., Underwood A., Lettini A.A., Page A. (2016). Microevolution of Monophasic Salmonella Typhimurium during Epidemic, United Kingdom, 2005–2010. Emerg. Infect. Dis..

[28] Switt A.I., Soyer Y., Warnick L.D., Wiedmann M. (2009). Emergence, distribution, and molecular and phenotypic characteristics of Salmonella enterica serotype 4,5,12:i. Foodborne Pathog. Dis..

[29] Hauser E., Tietze E., Helmuth R., Junker E., Blank K., Prager R., Rabsch W., Appel B., Fruth A., Malorny B. (2010). Pork contaminated with Salmonella enterica serovar 4,[5],12:i:-, an emerging health risk for humans. Appl. Environ. Microbiol..

[30] Gossner C.M., van Cauteren D., Le Hello S., Weill F.X., Terrien E., Tessier S., Janin C., Brisabois A., Dusch V., Vaillant V. (2012). Nationwide outbreak of Salmonella enterica serotype 4,[5],12:i:- infection associated with consumption of dried pork sausage, France, November to December 2011. Euro Surveill.

[31] Giacometti F., Bonilauri P., Serraino A., Peli A., Amatiste S., Arrigoni N., Bianchi M., Bilei S., Cascone G., Comin D. (2013). Four-year monitoring of foodborne pathogens in raw milk sold by vending machines in Italy. J. Food Prot..

[32] Grimont P.A.D., Weill F.X. (2007). Antigenic Formulae of the Salmonella Serovars.

[33] Tennant S.M., Diallo S., Levy H., Livio S., Sow S.O., Tapia M., Fields P.I., Mikoleit M., Tamboura B., Kotloff K.L. (2010). Identification by PCR of non-typhoidal Salmonella enterica serovars associated with invasive infections among febrile patients in Mali. PLoS Negl. Trop. Dis..

[34] EUCAST—European Committee on Antimicrobial Susceptibility Testing. https://www.eucast.org/.

[35] EUCAST Guidelines for Detection of Resistance Mechanisms and Specific Resistances of Clinical and/or Epidemiological Importance. https://www.eucast.org/fileadmin/src/media/PDFs/EUCAST_files/Resistance_mechanisms/EUCAST_detection_of_resistance_mechanisms_170711.pdf.

[36] Centre for Disease Control and Prevention Standard Operating Procedure for PulseNet PFGE of Escherichia coli O157:H7, Escherichia coli non-O157 (STEC), *Salmonella* serotypes, *Shigella sonnei* and *Shigella flexneri* (PNL05). https://www.cdc.gov/pulsenet/pdf/ecoli-shigella-salmonella-pfge-protocol-508c.pdf.

